# Assessment of Potential Targets for Deep Brain Stimulation in Patients With Alzheimer’s Disease

**DOI:** 10.14740/jocmr2127w

**Published:** 2015-05-08

**Authors:** Mayur Sharma, Milind Deogaonkar, Ali Rezai

**Affiliations:** a Center of Neuromodulation, Wexner Medical Center, The Ohio State University, Columbus, OH 43210, USA

**Keywords:** Deep brain stimulation, Neuromodulation, Alzheimer’s disease

## Abstract

Alzheimer’s disease (AD) is a progressive neurodegenerative disorder affecting 36 million people worldwide and 5.2 million in the United States. The pathogenesis of AD is still elusive. Accumulations of abnormal proteins (beta amyloid and tau protein), inflammatory cascades, abnormal responses to oxidative stress and alteration in oxidative metabolism have been implicated in AD. There are few effective therapeutic options available for this disorder at present. Neuromodulation offers a novel treatment modality for patients with AD. The databases of Medline and PubMed were searched for various studies in English literature describing the deep brain stimulation (DBS) in patients with AD. Various animal and human clinical studies have shown promising initial results with bilateral DBS targeting various anatomical nodes. In this review, we attempt to highlight the pathophysiology, neural circuitry and potential neuromodulation options in patients with AD. In appropriately selected patients, DBS can potentially delay the cognitive decline, enhance memory functions and can improve the overall quality of life. However, further randomized controlled trials are required to validate the efficacy of neuromodulation and to determine the most optimal target for AD.

## Introduction

Alzheimer’s disease (AD) is a progressive neurodegenerative disorder. This disorder was first described by Dr. Alois Alzheimer in 1901 in a 51-year-old woman with progressive memory loss [1]. AD is a leading cause of cognitive disability and dementia worldwide and in the United States. The number of people affected by AD has increased from 26.6 million in 2006 to 36 million in 2014 worldwide [2, 3] (http://www.alzheimers.net/resources/alzheimers-statistics/). Of these 36 million AD patients, 5.2 million are in the United States, of which 200,000 patients are less than 65 years of age (young onset AD). A new patient is diagnosed with AD in the United States every 67 s (http://www.alz.org/alzheimers_disease_facts_and_figures.asp#prevalence). In 2014, the healthcare expenditure on AD has been estimated to be 605 billion US dollars worldwide and 150 billion US dollars in the United States [3]. This enormous burden of AD poses significant challenges to the health care providers and the caregivers.

The current management options for AD include medications aimed at potentiating the cholinergic pathways (acetyl cholinesterase inhibitors) and mitigating the glutaminergic pathways (N-methyl D-aspartate receptors blockers) [4-6]. Anti-oxidant such as vitamin E has also been used as medical therapy for AD. These medications provide modest symptomatic benefits in patients with AD, without altering the course of disease [6]. The lack of efficacy and adverse effects of current medications led to exploration of alternative treatment modalities for AD.

Following the success of deep brain stimulation (DBS) surgery in a variety of movement and psychiatric disorders [7], this treatment modality has been explored as a potential therapeutic option in patients with AD [5, 8]. In this review, we attempt to highlight the pathophysiology, neural circuitry and potential neuromodulation options in patients with AD.

## Pathophysiology and Neural Substrate of AD

The etiopathogenesis of AD is not completely elucidated. Based on animal and human studies, a variety of pathogenic mechanisms such as accumulations of abnormal proteins (beta amyloid and tau protein), inflammatory cascades, abnormal responses to oxidative stress and alteration in oxidative metabolism have been implicated in AD [2, 3, 5, 9]. At molecular level, accumulation of beta amyloid protein and tau protein in different regions of the brain has been implicated in the loss of synaptic functions, defective metabolism, impaired cellular repair, cell death and thus development of AD. Hyperphosphorylation of tau protein (microtubule associated protein) leads to the formation of microtubule or neurofibrillary tangles which impair the cellular tubular transport mechanism and thus leads to neuronal death [5]. Accumulation of beta amyloid plaques has also been implicated in the pathogenesis of AD. Amyloid precursor protein (APP) with genetic loci on chromosome 21 binds to death receptor 6 and initiates the apoptotic pathways resulting in neuronal loss [5, 9, 10]. This hypothesis supports an earlier onset of AD in patients with trisomy 21. Accumulation of other proteins such as presenilin 1, presenilin 2 and apolipoprotein E4/E4 with genetic loci on chromosomes 14, 1 and 9 respectively has also been implicated in the pathogenesis of AD [3, 10].

These molecular alterations lead to neuronal loss and cerebral atrophy in the different regions of the brain involving frontal, temporal, parietal, hippocampus and entorhinal cortex (EC) [3, 5]. In addition to localized cellular loss, AD also disrupts the neuronal connections between the cortical and subcortical areas [8]. These neuronal connections are referred as default mode networks (DMN) and functional alterations in these networks have been linked to the memory deficits in patients with AD [8, 11]. Thus AD can be visualized as a systemic disorder affecting several neuronal networks instead of a localized neurodegenerative disorder. In a recent study, approximately one-third of AD cases worldwide have been attributed to potentially modifiable risk factors such as improved education status, vascular risk factors and depression [12].

## Literature Search

The databases of Medline and PubMed were searched for various studies in English literature describing the DBS in patients with AD. All related studies published up to December 2014 were included in this review.

## Animal Models of AD

Various rodent models have been used to demonstrate the efficacy of DBS in modulating the memory circuits and enhancing the neurogenesis [13, 14]. Hamani et al [14] showed that DBS of the anterior thalamic region in corticosterone-treated rats improved performance and enhanced hippocampal neurogenesis 1 month following stimulation. They postulated that long-term plastic changes and newly formed mature dentate gyrus cells are implicated in such positive findings [14]. Bilateral DBS of the anterior thalamic nucleus has been shown to reversibly increase glucose uptake in the thalamus/hippocampus and decrease uptake in the cingulate and frontal cortex in non-epileptic rats [15]. Decreased glucose uptake in thalamus/hippocampal region was observed following bilateral lesioning of anterior thalamic nuclei. Another animal study using TgCRND8 mice demonstrated that DBS (high frequency) of the midline thalamic region enhanced short-term memory in the CA1 region of hippocampus and also increased the non-amyloidogenic α-secretase activity [13]. These promising results instigated researchers for the clinical trials of DBS for modulating the memory circuits. At present there are six clinical trials of DBS in patients with AD including one at our center [3].

## Current DBS Targets Used in Patients With AD

DBS is a well-established therapy for a variety of medical refractory movement disorders (Parkinson disease (PD), essential tremors and dystonia) with over 100,000 implants worldwide [7]. DBS was first approved by Food and Drug Administration (FDA) for essential tremors in 1997 [16, 17]. Following the success of DBS in refractory movement disorders with more than 20 years of safety records, this modality has been investigated for psychiatric disorders (obsessive-compulsive disorders, major depression, eating disorders, and addiction), traumatic brain injury, post-traumatic stress disorder, Tourette’s syndrome, epilepsy, cluster headache, post-stroke pain, amputation pain, neuropathic pain, multiple sclerosis tremor, and impaired conscious state including dementias with varied success [18-28].

### Hypothalamic/fornix

Based on promising results in animal models, various targets have been explored as potential DBS targets to modulate and enhance the memory functions in patients with AD. Hamani et al [29] reported unexpected improvement in autobiographical memories in a patient following bilateral hypothalamic DBS for morbid obesity. DBS increased recollection indicating potentiation of memory circuits involving hippocampus and mesial temporal lobe (limbic circuits) [29]. Based on this initial report, Laxton et al [28] first conducted the phase 1 trial of DBS of hypothalamus/fornix in patients with AD. In this study, six patients with mild AD underwent bilateral chronic constant stimulation of hypothalamus and the anterior border of vertical portion of fornix for 12 months. Monopolar stimulation was applied at 130 Hz, 90 μs pulse width and increasing the voltage by 1.0 V every 30 s until side effect is observed or a maximum intensity of 10 V (3 - 3.5 V). Based on AD assessment scale cognitive subscale and the mini-mental state examination (MMSE) scale, two of six patients showed slower rate of cognitive decline and one patient showed improvement in cognitive functions at 6 and 12 months. PET-CT scans following 12 months of continuous stimulation also demonstrated reversal of impaired glucose metabolism in the temporal and parietal lobes in this study [28]. No serious adverse events were noted in this study. Improved glucose metabolism in the cortical and hippocampal circuits following bilateral DBS of fornix at 1 year using PET-CT scans has been demonstrated in five patients with mild AD in an open label study [30]. Increased glucose metabolism was observed in frontal-temporal-parietal-striatal-thalamic circuit and frontal-temporal-parietal-occipital-hippocampal circuit which correlated with improvement in global cognitive functions and quality of life [30]. Fontaine et al [31] demonstrated stabilization of memory scores and increased metabolism in the mesiotemporal lobe following bilateral DBS of fornix in a patient with mild to moderate AD at 52 months. Bilateral low frequency stimulation of fornix using in-depth electrodes has been shown to improve MMSE in 11 patients with intractable epilepsy over a period of 4 h [32]. A major caveat is that these are non-randomized studies without placebo control and therefore results should be interpreted with caution.

### Nucleus basalis of Meyernet (NBM)

Based on animal studies [33], NBM has been explored as a potential target for DBS in patients with dementia [34]. Freund et al [34] implanted bilateral NBM DBS in addition to subthalamic nucleus (STN) DBS in a 71-year-old patient with PD-related dementia. Improvement in attention, alertness, drive, concentration and spontaneity was observed following bilateral NBM stimulation. This improvement in cognitive and behavior functions can be attributed to the potentiation of residual cholinergic projections in the memory circuits [34].

### EC/hippocampus

Since EC/hippocampus is an integral part of Papez or memory circuits, modulation of these structures can be explored as potential therapeutic targets in patients with AD. Fell et al [35] conducted a pilot study to evaluate the effects of low frequency stimulation of EC and hippocampus in 11 patients with temporal lobe epilepsy. They reported a linear correlation of stimulation on correctly remembered words. The best response was seen with in-phase stimulation followed by sham and least with anti-phase stimulation [35]. Another study demonstrated enhancement of spatial memory functions with resetting of theta rhythm on EEG, following in-depth stimulation of EC during learning in seven patients with refractory epilepsy [36]. These studies support the connections between the EC and hippocampus and their role in modulating the memory or learning processes.

### Pedunculopontine tegmental nucleus (PPN)

Stefani et al [37] implanted bilateral PPN DBS in addition to STN DBS in six patients with advanced PD-related dementia. Low frequency stimulation (25 Hz) of the PPN improved both attention and executive functions. Increased glucose metabolism was noted in the bilateral frontal, prefrontal cortical areas and left ventral striatum following stimulation. Stimulation parameters used in this study were 2.4 V, 25 Hz frequency and 60 μs pulse width. This study showed that PPN DBS has a potentiating effect on both associative and limbic pathways and can be a potential target for DBS in patients with AD.

### Anterior limb of internal capsule/nucleus accumbens (ALIC/NAc)

Ventral capsule provides a conduit for the white matter tracts originating from the basal ganglia, brain stem and hippocampus and projecting to the frontal lobe. Ventral striatum and nucleus accumbens has also been implicated in motivation and is a part of reward circuitry [38]. Modulation of these pathways has been shown to have beneficial effects in a variety of disorders including obsessive compulsive disorders, depression, obesity, addiction and anxiety disorders [7, 25, 38]. Therefore, modulating these neural circuits might improve the cognitive and behavior functions in patients in AD. The initial results of the study involving bilateral DBS of ALIC/NAc in patients with AD are promising and yet to be published.

## Conclusion

AD is a complex disease with significant financial and healthcare burden. The medical therapy for AD is of modest benefit and associated with significant side effects. The use of neuromodulation in the treatment of AD is a relatively newer modality and the most optimal target is yet to be elucidated. DBS of the fornix/hypothalamus has shown promising results in terms of both delaying and reversing the cognitive deterioration in patients with AD. Other targets such as NBM, PPN, ALIC/NAc and EC have been explored with good initial results. In appropriately selected patients, DBS can potentially delay the cognitive decline, enhance memory functions and can improve the overall quality of life. However, long-term randomized controlled trials are required to validate the efficacy of neuromodulation and to determine the most optimal target for AD.

## Abbreviations

DBS: deep brain stimulator
AD: Alzheimer disease
APP: amyloid precursor protein
NBM: nucleus basalis of Meyernet
PPN: pedunculopontine nucleus
ALIC/NAc: anterior limb of internal capsule/nucleus accumbens
